# A Video-Delivered Family Therapeutic Intervention for Perinatal Women With Clinically Significant Depressive Symptoms and Family Conflict: Indicators of Feasibility and Acceptability

**DOI:** 10.2196/41697

**Published:** 2022-10-04

**Authors:** Fallon Cluxton-Keller, Mark T Hegel

**Affiliations:** 1 Department of Psychiatry Geisel School of Medicine Dartmouth College Lebanon, NH United States

**Keywords:** family intervention, perinatal, postnatal, depression, conflict, telehealth, family conflict, family therapy, family therapist, video conferencing, teleconference, teleconferencing, telemedicine, virtual care, mental health, psychological health, digital health intervention, parenting

## Abstract

**Background:**

Variation in family therapeutic intervention fidelity has an impact on outcomes. The use of video conferencing technology can strengthen therapist fidelity to family therapeutic interventions.

**Objective:**

This article explores indicators of feasibility and acceptability for a video-delivered family therapeutic intervention for perinatal women with depressive symptoms and family conflict. The objectives of this article are to describe indicators of feasibility, including therapist fidelity to the intervention and technological factors that relate to implementation of the intervention, as well as indicators of acceptability for participants of the intervention.

**Methods:**

The data included in this article are from an ongoing randomized trial of the Resilience Enhancement Skills Training (REST) video-delivered family therapeutic intervention. Participant recruitment and data collection are still underway for this clinical trial. Of the 106 participants who are currently enrolled in this study, 54 (51%) have been randomized to receive REST from May 2021 through July 2022. Currently, 2 therapists are delivering the intervention, and the training procedures for therapists are summarized herein. Therapist fidelity to the family therapeutic intervention was assessed in 67 audio recorded sessions. The training procedures were summarized for use of video conferencing technology by therapists and the 54 study participants. Knowledge of the video conferencing technology features was assessed in therapists and study participants by the number of attempts required to use the features. Participant responsiveness to the intervention was assessed by the percentage of attended sessions and percentage of complete homework assignments.

**Results:**

To date, both therapists have demonstrated high fidelity to the family therapeutic intervention and used all video conferencing technology features on their first attempt. The current participants required 1 to 3 attempts to use 1 or more of the video conferencing technology features. About 59% (n=32) of the current participants immediately accessed the features on the first attempt. Our results show that perinatal women attended all sessions, and their family members attended 80% of the sessions. To date, participants have completed 80% of the homework assignments.

**Conclusions:**

These early findings describe indicators of the feasibility and acceptability of the video-delivered family therapeutic intervention for use with this high priority population. Upon completion of recruitment and data collection, a subsequent article will include a mixed methods process evaluation of the feasibility and acceptability of the video-delivered family therapeutic intervention.

**Trial Registration:**

ClinicalTrials.gov NCT04741776; https://clinicaltrials.gov/ct2/show/NCT04741776

## Introduction

### Background

The federal home visiting program is a voluntary program that provides parenting education to pregnant and postpartum women and their families [1]. Several studies have shown that over a third of home visited mothers (pregnant and postpartum) report clinically significant depressive symptoms [2-4]. Home visited mothers infrequently obtain treatment due to barriers (eg, lack of childcare, lack of transportation, stigma [4,5]) that are significantly greater in rural regions. Nonviolent family conflict, defined as expressed anger, criticism, and arguments [6] worsens depressive symptoms in perinatal women [7-10]. Yet, the research is limited on family therapy interventions that address depressive symptoms in perinatal populations with family conflict [11-13].

The use of HIPAA (Health Insurance Portability and Accountability Act)-compliant video conferencing technology (VCT) to deliver family therapeutic interventions that address perinatal depressive symptoms and family conflict may be a feasible strategy to increase access to treatment. However, research is limited on the feasibility of using VCT to deliver family therapeutic interventions to this population [11-13]. While the use of VCT is not new to the field of family therapy, most studies have examined the feasibility and effectiveness of interventions that primarily target parenting skills for youth with specific mental health problems (eg, [14-18]).

The rapid shift from in-person to VCT sessions during the COVID-19 pandemic created fidelity challenges in the implementation of many family therapy models that address family conflict [eg, 17]. Variation in intervention fidelity can have unintended impacts on outcomes [19,20]. Implementation science research shows that several factors (eg, therapist training, frequency of supervision, etc) contribute to intervention fidelity [19,20]. Technology influences how the therapist and family interact in sessions, which can impact adherence to the model and family outcomes [16]. Therapists who lack specific guidance on delivering models using VCT may experience problems with delivering the model as intended. For example, low bandwidth can lead to frustration in therapists and families due to having to repeat themselves, as well as omission of some session content due to reduced audio quality [18]. Thus, model developers that aim to use VCT for session delivery need to include specific steps for therapists to manage technological challenges that could interfere with session quality.

Concordantly, families need to be trained in how to use VCT to participate in the sessions. A recent systematic review [16] on the use of VCT for couple and family therapies showed that of the 37 included studies, only 3 [12,21,22] reported that families were specifically trained in the use of the VCT. Given that many families seek family therapy to address conflict, lack of clarity on how to use VCT to join the session could lead to arguments before the session even starts or cause them to miss sessions.

This study builds on prior research of a video-delivered family therapeutic intervention called Resilience Enhancement Skills Training (REST) [12,23], which aims to reduce perinatal depressive symptoms and family conflict in home visited families in rural areas. REST is based on general systems theory [24] and informed by family-oriented Dialectical Behavior Therapy Skills Training [25]. REST is not a parenting skills program. It was developed prior to the COVID-19 pandemic and leverages VCT to increase access to treatment [12]. Implementation of REST requires training of the therapist and families to ensure correct use of VCT in sessions [12].

### Objectives

This article explores indicators of feasibility and acceptability from an ongoing randomized trial of REST. The article has 2 objectives: the first is to describe 2 indicators of feasibility that include therapist fidelity to REST and technology factors relating to the implementation of REST, and the second is to describe 2 indicators of acceptability that include session attendance and homework completion.

## Methods

### Study Design

This ongoing study uses an effectiveness-implementation hybrid type 1 design with a pilot randomized trial of REST compared to standard of care video-delivered problem solving therapy (V-PST) (eg, [26]). Mothers are randomly assigned to receive REST or V-PST. Both interventions are delivered using VCT and include a total of 10 weekly 45-minute sessions. The mother participates in REST sessions with a family member, whereas the mother participates alone in the V-PST sessions. This article focuses on indicators of feasibility and acceptability of REST and only uses the current data from that arm of the trial.

### Ethics Approval

This trial was approved by an institutional review board (STUDY02000691) at an academic medical center in New England. Study participants provided consent for the eligibility screen and study enrollment, including consent for audio recorded sessions.

### Participant Eligibility

Each family consists of the mother and her family member, defined as her adult relative or current intimate partner. Based on information from the participating home visiting agencies that most mothers had 1 eligible family member, we limited the number of family members to just 1. A research team member schedules separate phone calls with the mother and her family member to obtain electronic consent for the eligibility screen interview. The eligibility screen interview phone call is done separately with each mother and her family member because the interview for mothers includes the Abuse Assessment Screen [27] to screen for domestic violence, and family conflict is assessed separately using the Perceived Hostility Survey (PHS) [28].

Mothers are eligible for participation in this study if they meet the following inclusion criteria: (1) enrolled in home visiting at a participating agency; (2) in any trimester of pregnancy and up to 18 months postpartum; (3) at least 15 years old; (4) fluent in English with at least an eighth-grade education, as intervention materials are written in English for this level of education; (5) experiencing moderate-to-severe depressive symptoms, with scores of at least 20 without suicidal ideation on the Beck Depression Inventory-Second Edition (BDI-II, [29]); (6) experiencing moderate to high conflict (PHS ages 18 + raw scores of at least 16; PHS ages 15-17 years old raw scores of at least 14) [30] with the selected family member with whom they live in the same home or have at least weekly contact; and (7) have consistent internet access on a cell phone, tablet, or computer with a working camera and microphone. This study includes a detailed protocol for mothers who report suicidal ideation on the BDI-II (rating of 2 or 3 on item 9), and they are provided with emergency assistance. Mothers who report current domestic violence in their homes or histories of domestic violence with the selected family member on the Abuse Assessment Screen are ineligible for participation in this study. This study includes a detailed protocol for mothers who report domestic violence, and they are provided with emergency assistance by local service providers who address domestic violence.

Family members are eligible for participation if they meet the following inclusion criteria: (1) are the mother’s adult relative or current intimate partner; (2) at least 15 years old; (3) fluent in English with at least an eighth-grade education, as intervention materials are written in English for this level of education; 4) experiencing moderate to high family conflict with the mother (PHS ages 18 + raw scores of at least 16; PHS ages 15-17 years old raw scores of at least 14 [30]); and (5) have consistent internet access on a cell phone, tablet, or computer with a working camera and microphone.

### Recruitment

Participants are recruited from participating home visiting agencies that serve low-income families in New England. Home visitors use depression screening and referral procedures to refer mothers to the study [12]. Participant recruitment began in April 2021 and is still underway. The goal is to achieve a sample size of 160 individuals (80 families). As of July 2022, 106 participants have enrolled in the study, and 54 (51%) of them are assigned to REST.

### Overview of REST

REST includes a total of 10 weekly 45-minute sessions delivered by a licensed mental health professional using VCT. In this study, 2 masters-level licensed social workers are currently delivering REST to participants. REST is based on general systems theory [24] and informed by family-oriented Dialectical Behavior Therapy (DBT) skills training [25]. REST uses interventions informed by general systems theory [24] to change dynamics that produce family conflict. REST is informed by DBT core skills to improve regulation in cognition (mindfulness), emotions (behavioral activation and cognitive restructuring), and behavior (boundaries) [25]. Similar to DBT skills training [25], REST uses a psychoeducational format to teach skills to families. Most families consider REST sessions to be educational classes.

REST requires the therapist’s use of 2 documents that pertain to session content and quality: (1) the REST therapist manual (including safety protocols for depression and family conflict) and (2) the skills book for families. The therapist asks participants to select up to 3 goals that pertain to their relationship and mood in the first session. Similar to DBT skills training [25], REST includes sessions that are devoted to teaching participants new skills and some that are devoted to reviewing learned skills. The skill introduction sessions require the therapist to use the screen share feature in the VCT (which allows participants to view a document on the therapist’s computer screen during the session) to read content from the skills book on the purpose and use of the skill to participants. All sessions include a mindfulness exercise, a session goal that includes a summary of the activities, and an explanation of the ways in which the skills relate to the participants’ goals. Nearly all sessions include skill practice exercises. Therapists verbally communicate participant strengths in at least 8 sessions. Nine sessions include planned skill application questions to help participants prepare for homework assignments. The skill application elements of REST are flexible and allow therapists to work with families to apply the skills to aspects of their relationships based on each family’s preferences. Participants receive homework assignments in 9 sessions. Although REST was originally designed for families, we anticipated that some family members would miss sessions. For this reason, practice exercises that involve role-plays and some of the skill application questions were modified for use in sessions only attended by the mother.

### Training Procedures

#### REST Training

The primary aim of REST training is to teach therapists how to deliver session content to participants. The first author (FC-K) trained both therapists in the implementation of REST during a 6-hour virtual training session. The training session included 2 parts: (1) 2 hours of didactic instruction on the REST therapist manual and practice exercises for the skills, and (2) 4 hours of role-plays with feedback to allow therapists to demonstrate use of the REST therapist manual for skill application for cognitive, emotion, and behavior regulation in different family scenarios. Role-plays were conducted so that therapists could practice delivering REST using VCT. The first author then provided feedback to therapists during role-plays. Supervision sessions are devoted to reinforcing content from the 6-hour training session. The first author supervises each REST therapist separately on a weekly basis using VCT. Results from ongoing fidelity assessments are used to continuously monitor their adherence to the model, and corrective feedback is provided as needed.

#### Technology Training

REST requires therapists and participants to use VCT for sessions. WebEx (Cisco) [31] is the VCT used to deliver REST in this study. The technology requirements for therapists include a computer with a working microphone and camera, a word processing program to open the skills book, and consistent, secure internet access (eg, ethernet connection) with a WebEx account. Technology requirements for participants include consistent internet access (eg, subscription to an internet service provider) on an electronic device (eg, cell phone, tablet, or computer) with a working camera and microphone, along with the WebEx app to participate in sessions.

Each therapist underwent a separate training session to learn how to download and install the VCT software on the computer and use all features, including scheduling sessions, audio, video, screen share, and audio recording. One therapist required a single 15-minute training session and the other required a single 30-minute training session on the use of VCT. The second therapist’s training was longer because she had recently purchased a new laptop computer and was still in the process of learning how to use it. In this study, 1 therapist used the split screen feature on her computer to view the REST therapist manual on one side of the screen and the skills book for the screen share for participants on the other side. The other therapist did not want to use the split screen feature and chose to place a hard copy of the REST therapist manual on a stand beside her computer and the skills book content on her computer for the screen share element in sessions.

Each participant received an individualized training session on use of the VCT for sessions. In this study, the first author trained the participants in use of the VCT. Each participant was emailed a link to join the VCT demonstration meeting and instructed to click the link to download and install the VCT app on the selected device (cell phone, tablet, or computer). Next, the participant followed the prompts to log in to join the meeting. When the participant joined the meeting, instructions were provided to click the microphone icon for audio and the camera icon for video.

Therapists received ongoing guidance on managing technology challenges that could occur in sessions. Problems with low bandwidth can result in poor audio quality. In these instances, therapists were directed to turn off the video feature and to ask participants to turn off theirs as well. Participants could still see the therapist’s screen shared document when the videos were turned off. Turning off the video worked adequately in nearly all instances when low bandwidth was the problem. In the limited instances that turning off the video did not improve the audio quality, therapists were directed to use the phone call feature in the VCT and continue to use the video feature for only the screen shares in sessions. In the rare instance of significant internet service interruptions (eg, power outage due to a storm), therapists were instructed to continue the sessions using only the phone call feature in the VCT. Additional guidance was provided to therapists after we discovered that some participants with specific types of cell phones experienced significant reduced audio quality (low volume and choppy audio) when they used the ignore feature to avoid incoming phone calls during sessions. In these instances, therapists instructed participants to exit the session and rejoin it.

### Data Collection

Research staff verbally administered the baseline questionnaires to mothers and family members, separately, prior to the first session and entered the data in REDCap (Research Electronic Data Capture) [32,33]. In this article, there are 2 indicators of feasibility, which include (1) REST fidelity, defined as the therapist’s knowledge of REST and adherence to session content [34], and (2) knowledge of VCT in therapists and participants. The 2 indicators of acceptability center on participant responsiveness and include (1) session attendance and (2) homework completion [34].

Fidelity was assessed by therapist knowledge of and adherence to the REST therapist manual. For therapist knowledge of REST, the first author (FC-K) rated therapists’ knowledge of the cognitive, emotion, and behavior regulation skills on the REST knowledge test during the role-plays during the REST training. Therapists were not allowed to deliver REST until they earned 100% on the test. To monitor therapist fidelity to REST session content in the ongoing trial, sessions are audio recorded. The first author developed the REST fidelity measure to rate therapist adherence to REST session content in the audio recorded sessions, and both authors (FC-K and MTH) use the measure to monitor fidelity in this study. Therapists are rated on the quality of content delivery for each session. For all sessions, therapists are rated in content categories on a scale that ranges from 0 to 2 (0: no content delivered, 1: partial content delivered, and 2: all content delivered). The maximum number of points for each session varies depending on the skill content. The level of fidelity is assessed to ensure that each therapist achieves at least 80% on the REST fidelity measure for each session, and data from these ongoing assessments are used in weekly supervision.

The current trial includes a 2-phase fidelity monitoring process for REST. In phase 1, both authors assessed fidelity in audio recorded sessions for the first 2 families assigned to each REST therapist. Fidelity was assessed following each session and before the next scheduled session so that the first author could immediately provide corrective feedback to REST therapists as needed. Both authors discussed and resolved any discrepancies in these fidelity assessments.

In phase 2, the second author (MTH) assesses fidelity in 10% of randomly selected audio recorded REST sessions that were originally assessed by the first author. Phase 2 is ongoing, as this study is still underway. The level of fidelity is continuously assessed to ensure that each therapist achieves at least 80% adherence to REST with each family. Both authors discuss and resolve any discrepancies in the fidelity assessments as needed.

The first author assessed therapist knowledge of VCT by observing their use of the audio, video, screen share, and recording features on their computers during the training. The first author also assessed participant knowledge of VCT by observing whether they joined the demonstration meeting and used the audio and video features on their electronic device (cell phone, tablet, or computer).

For participant responsiveness, data were collected on participant session attendance and homework completion from the therapists’ session notes. Therapists were instructed to document attendance and homework completion in the session notes. The session notes do not contain identifying information about the participants, and all notes are stored on a password-protected, secure site within the medical center network.

### Analytic Plan

Univariate statistics are used to characterize REST participants’ demographic and psychosocial information. This article includes demographic characteristics of participants who enrolled in the trial and were randomized to REST from May 2021 to July 2022.

For therapist knowledge of REST, the first author calculated the number of times it took each therapist to demonstrate correct use of the REST skills during role-plays. For adherence, Cohen Kappa was used to assess inter-rater reliability of the REST fidelity measure. A κ value of at least 0.80 suggests sufficient inter-rater reliability [35]. The mean adherence score (percent) for delivery of session content was calculated using the average adherence score divided by the total possible score. The item mean was calculated for adherence to REST session components, and the percentage for all content delivered (rating of 2 on the REST fidelity measure) was calculated for each component. The number of sessions that included lack of adherence to REST and deviations were calculated, and the reasons were recorded.

For knowledge of VCT, univariate statistics were used to summarize the number of attempts needed by each therapist and study participant to access and demonstrate use of the audio and video features in the VCT. For therapists only, the number of attempts needed to correctly use the screen share and audio recording features in the VCT were calculated. For study participants only, the number of attempts needed to join the VCT demonstration meeting were calculated.

For participant responsiveness, the total number of attended sessions were calculated separately for mothers and their family members. The proportion of attended sessions to expected sessions (total of 10) was calculated. The proportion of completed homework assignments to expected homework assignments (total of 9) was calculated.

## Results

### Therapist Characteristics

Both therapists are female licensed social workers with over 10 years of experience working with home visited families. One therapist identifies as more than 1 race, and the other identifies as Caucasian. Both therapists have knowledge of general systems theory and cognitive-behavioral family therapies.

### Participant Characteristics

This trial is ongoing, and recruitment and data collection are still underway. For the purpose of this article, this section describes the demographic and psychosocial characteristics at baseline for the 54 participants who enrolled in the study and were randomly assigned to REST from May 2021 to July 2022. The race representation of the 54 participants included 72% (n=39) White non-Hispanic, 11% (n=6) more than 1 race, 9% (n=5) Asian, 4% (n=2) American Indian, and 4% (n=2) Black or African American. For highest level of education, 20% (n=11) of participants reported they did not graduate from high school, 43% (n=23) had high school degrees, 17% (n=9) had some college but no degree, and 20% (n=11) had higher education degrees. About 89% (n=48) of the family members were the mother’s current intimate partner. On average, mothers were 26.35 (SD 4.90) years old, and their family members were 32.15 (SD 13.20) years old at baseline. Of the 27 mothers assigned to REST, about 15% (n=4) were pregnant at baseline. The baseline mean BDI-II score for mothers was 29.85 (SD 5.69), indicative of severe depressive symptoms. The baseline mean PHS uncorrected *T*-score for mothers was 58.7, indicative of moderately high conflict with family members. The baseline mean PHS uncorrected *T*-score for family members was 60.1, indicative of high conflict with mothers.

### Feasibility

#### Fidelity

Each therapist earned 100% on the REST knowledge test on the first attempt in demonstration of cognitive, emotion, and behavior regulation skills in role-plays. Both authors assessed therapist adherence to REST session content using the REST fidelity measure. The authors established sufficient inter-rater reliability (κ=0.91) on the REST fidelity measure for the 67 audio recorded sessions. The average session length was 43 (SD 17.07) minutes. Three families experienced significant childcare interruptions, which extended the length of some sessions by 15 to 30 minutes.

One therapist achieved 90% adherence to REST in the sessions with the first 2 families to which she was assigned. She did not deliver some content in a few sessions (described below), but on average, she has adhered to 90% of REST content in the 32 audio recorded sessions assessed by the authors. As previously mentioned, the other therapist experienced some technological problems with her computer in sessions with the first family to which she was assigned, which resulted an adherence score that fell below 80% in the first 4 sessions. In these instances, she delivered content to the family that was missed in subsequent sessions. After her computer was fixed, she achieved 90% adherence to REST in sessions with the second and third families to which she was assigned. She did not deliver some content in a few sessions (described below), but on average, she has adhered to 88% of REST content in 35 audio recorded sessions assessed by the authors. Table 1 includes the average adherence for all content by session number and number of audio recordings assessed by the authors.

Table 2 includes the average adherence to REST session components for both therapists combined.

**Table 1:** Therapist adherence to Resilience Enhancement Skills Training (REST) by session number.

Session by audio recordings, n	Overall adherence to session content, %
Session 1: 10	99
Session 2: 7	94
Session 3: 8	92
Session 4: 5	100
Session 5: 7	89
Session 6: 6	92
Session 7: 4	92
Session 8: 7	92
Session 9: 7	99
Session 10: 6	94

**Table 2:** Therapist adherence to Resilience Enhancement Skills Training (REST) by session content.

Component by audio recorded sessions, n	Delivery of all content, %
Mindfulness exercise: 67	93
Homework review: 57	97
Session goal: 67	90
Purpose of skill^a^: 50	92
Explanation of how skill relates to participant goal: 67	87
Practice exercise: 61	94
Planned skill application: 61	82
Homework assignment: 61	95
Verbalize family strength: 57	88

^a^No variation by skill type.

Both therapists did not deliver some content in 7 sessions (1 session per family), which resulted in session adherence scores below 80%. Lack of adherence to content occurred in 4 sessions when participants arrived over 15 minutes late, and the therapists skipped the explanation of how the skills related to a participant goal, did not verbalize a participant strength, or partially delivered the planned skill application content due to time limitations. To prevent this from occurring again, the authors informed both therapists to reschedule the session if they have back-to-back sessions scheduled and a family does not arrive within 15 minutes of the set time. Lack of adherence to the planned skill application content occurred in 3 sessions with mothers of older children who frequently interrupted sessions with requests for privileges (eg, requests to play video games).

Other minor deviations occurred in sessions, but therapists still achieved at least 80% adherence to the session content. For example, a total of 5 sessions included participants who were diagnosed with significant medical (nonpsychiatric) problems, and they processed their thoughts and feelings at the beginning of the sessions. In these instances, the therapists validated the participants’ thoughts and feelings, reinforced the importance of talking with their medical providers about medical treatment options, and redirected to them to the session content.

#### Knowledge of VCT

Both REST therapists demonstrated use of the audio, video, screen share, and audio recording features in the VCT on the first attempt during the training. One therapist initially had difficulty with consistently accessing the screen share feature in 4 sessions with the first family she served because the mouse pad on her laptop worked inconsistently. The COVID-19 pandemic caused a shipping delay for the external mouse that was compatible with her computer, but when she transitioned to the use of an external mouse, she did not experience any other technological problems in sessions. The therapists initially had some difficulties with the audio recording feature in the first session with a total of 3 families, in that they realized they were not audio recording about 10 minutes into the session.

On average, the training in use of VCT for participants was 10 minutes. All participants were able to download and install the VCT app on their devices on the first attempt. About 87% (n=47) of participants chose to install it on their cell phones, while 2% (n=1) chose to install it on a desktop computer, 4% (n=2) on a tablet, and 7% (n=4) on a laptop computer. About 70% (n=38) of participants immediately joined the demonstration meeting on the first attempt. Moreover, 59% (n=32) of participants immediately accessed the audio and video features on the first attempt. About 6% (n=3) participants required 2 attempts to access the audio feature, and 4% (n=2) participants required 2 attempts to access the video feature. Approximately 2% (n=1) of participants accessed the audio feature on the third attempt, and 24% (n=13) joined the demonstration meeting and accessed the audio and video features on the second attempt. About 6% (n=3) of participants joined the meeting and accessed the audio and video features on the third attempt.

To date, 9% (n=5) of participants who used cell phones to participate in the sessions experienced instances of low volume when they ignored incoming phone calls during sessions. The therapists asked the participants to exit the session and rejoin it, which resolved the issue in nearly all instances. In a total of 3 sessions, 2 participants used the phone call feature due to low bandwidth.

### Acceptability

#### Participant Responsiveness

To date, 31 participants have completed REST, and it is currently in progress for 18 participants. Of the 54 participants assigned to REST, 2 dropped out prior to the second session due to busy work schedules and 2 dropped out prior to the fourth session because they moved to a different state. One family member discontinued REST prior to the second session due to a need for intensive medical treatment.

To date, all 16 mothers have attended all 10 sessions. On average, family members attended 80% of the sessions. However, 33% (5 out of 15) of family members had to miss at least 1 session due to variable work schedules or increased work hours. The participants who completed REST also completed 80% of the homework assignments. COVID-19 and other physical illnesses in participants and their children were primary reasons why they did not complete homework assignments.

## Discussion

### Principal Results

Our findings suggest that we have identified feasible strategies to facilitate REST’s fidelity. REST is a highly structured psychoeducational intervention that requires the therapist to use basic technological skills (knowledge of VCT software features, average typing speed) to deliver it to families. These preliminary results suggest that the therapists have achieved high fidelity to REST. To date, the findings also suggest that REST is acceptable to families, in that mothers have attended all sessions and family members have attended 80% of sessions. Given the psychoeducational format of REST, participants consider sessions to be classes to learn new skills. To date, the participants who have completed REST have also completed 80% of the homework assignments.

Both therapists mastered use of the audio, video, screen share, and audio recording features in the VCT on the first attempt. About 70% of REST participants joined the VCT demonstration meeting on the first attempt. Over half (59%) of REST participants accessed the audio and video features in the VCT on the first attempt. To date, REST participants that required 2 or more attempts to access audio or video features in the training have been able to access these features on the first attempt to join the sessions. Therapists have been provided with specific guidelines to prevent and manage potential technology interruptions, which has been helpful in maintaining participant engagement in the sessions. The guideline that is most often used by therapists pertains to poor audio quality that occurs when participants mute incoming phone calls during sessions. To resolve this problem, therapists ask the participants to exit the session and rejoin it.

### Comparison With Prior Work

These early findings on the indicators of feasibility and acceptability of REST align with those of previous research on REST [12,23]. REST has been delivered using 2 types of VCT: Vidyo [36] in the preliminary study [12] and WebEx in this study. The early findings of this study suggest that masters-level therapists can deliver REST with high fidelity. To date, findings that pertain to session attendance are consistent with those previously reported in the preliminary pilot study of REST [12], in that mothers have attended all 10 sessions and family members have attended 80% of sessions. In the preliminary pilot study of REST [12,23], the therapist and home visitors reinforced participants’ use of the skills, but only therapists reinforced participants’ use of the skills in this trial. The decision to eliminate home visitor reinforcement of REST skills in this study was based on home visiting agency staff reports that they did not have enough time to complete this task during home visits. We do not believe this change has impacted the homework completion rates. Unlike the preliminary pilot study of REST [12], this study is being conducted during a pandemic, and some participants were unable to complete homework assignments due to COVID-19 and other physical illnesses in themselves or their children.

### Limitations

This study is ongoing, and more data are needed to gain a comprehensive knowledge of the feasibility and acceptability of REST. The study is currently in phase 2 of the fidelity monitoring plan, and a subsequent article will include the complete findings on therapist adherence to REST. Since recruitment and data collection are currently underway, outcome data that compare REST to the standard of care (V-PST) will be included in a future publication.

### Conclusions

This article explored indicators of feasibility and acceptability of REST. Evidence of feasibility and acceptability is important in justifying REST’s potential for scalability. Upon completion of data collection, a mixed methods process evaluation will be conducted to fully explore the feasibility and acceptability of REST. The findings on therapist fidelity and family perceptions of REST will be used to guide interpretation of REST’s preliminary effectiveness to inform a larger trial.

## Abbreviations

BDI-II: Beck Depression Inventory-Second Edition
DBT: Dialectical Behavior Therapy
HIPAA: Health Insurance Portability and Accountability Act
PHS: Perceived Hostility Survey
REDCap: Research Electronic Data Capture
REST: Resilience Enhancement Skills Training
VCT: video conferencing technology
V-PST: video-delivered problem solving therapy
